# Foreign Summary

**Published:** 1840-03-14

**Authors:** 


### FOREIGN SUMMARY.

*Dysmenorrhœa, accompanied by inflammatory congestion of the Cervix Uteri, effectually relieved by superficial scarification of that part.* By J. L. Fenner.—I am anxious to communicate the following case, and its treatment, as I have reason to believe that the scarification of the cervix uteri, in these painful cases, is nearly, if not entirely, an original suggestion, especially with regard to abstracting from it a definite quantity of blood. Dr. Ashwell saw the case with me, and was much pleased with its effects. He requested that the operation might be repeated as circumstances required.

Mrs. ——, a widow, æt. 39, had been long afflicted with dysmenorrhœa, accompanied with inflammatory congestion of the uterus, dating its origin many years since, from a severe and protracted labour. The nervous system was so entirely implicated in this affection, that the superior and inferior extremities, as well as the body, were continually agitated by a species of chorea. She was passing through a three months' course of mercurial friction, and had no relief from opium or any kind of narcotics. Leeches alone, applied round the cervix uteri, had palliated her sufferings, and these acted like enchantment, dissipating every symptom, and, after restless nights, producing a calm, refreshing sleep of some hours' duration.

*Nov.* 1, 1839.—Appreciating the relief obtained from the abstraction of blood and its tendency to remove congestion, it struck me as quite practicable, aided by my cylindrical tubular speculum (described in the Medical Gazette, May 25, 1839, and may be seen at instrument makers,) easily to abstract, by slight scarifications of the cervix uteri, any quantity I might think desirable. After a few superficial scarifications the blood trickled freely, and, in a quarter of an hour, two ounces and a half (by weight) were obtained, and the tube withdrawn, when the bleeding immediately ceased. Precisely the same relief followed with uninterrupted sleep, as was wont to result from the application of leeches. The patient said the operation was so painless that it would not even have disturbed her sleep. Dr. Ashwell saw the patient with me, and requested that the operation might be repeated, as it seemed to have been very beneficial.

2d.—Two ounces and a half of blood were obtained under the same circumstances.

3d.—Three ounces and a half of blood.

5th.—The cervix uteri having many marks of scarification, the tube was withdrawn a little, so as to expose the *cul de sac* of the vagina. Scarifications were made, presuming that it would bleed freely, because to that part of the vagina leeches have been applied by tubes perforated at the end with holes, and unscientifically thrust up the vagina; but such tubes cannot be duly applied to the cervix uteri, though sometimes to a portion of its side. The blood trickled freely, and in a quarter of an hour four ounces (by weight) were obtained, with the same relief as by leeches.

9th.—The patient having obtained more decided relief than on any former occasion from the application of leeches, the scarification is to be resumed as occasion may require, and the mercurial friction to be continued to the given time.

I have performed this operation on two other patients, and, since writing the above, have abstracted five ounces of blood (by weight) from the cervix uteri.—*Medical Gazette.*

---

*On the Treatment of Acute Rheumatism by Opium.* By D. J. Corrigan, M. D.—Eight cases of acute rheumatism cured by opium alone are given in detail by Dr. Corrigan, and as the practice appears to be novel, whilst it recommends itself from its simplicity and general applicability, a short notice of these cases may be read with interest. When he first commenced the opium treatment he was afraid to trust to it alone, and therefore combined it with calomel; but finding that no good resulted from this combination, he dropped the calomel, and continued the opium alone.

Three of the cases are selected to illustrate the mode in which the remedy was administered.

Mr. R. aged 30, was seen on the 19th of January, 1838. For several days he suffered from flying pains, but for the last three days he has been under the most acute suffering from pain in the shoulders, in the back of the neck, along the loins, in the knee, wrist, and ankle-joints. The knees, ankles, and wrists were swollen, and exquisitely painful. He had slept none for three nights; the pulse was one hundred and thirty-two; the bowels free; the tongue covered with a thick white coat; the urine very high-coloured, and depositing a pink sediment; and the skin partially perspiring. One grain of opium with two of calomel were given every third hour, and opiate fomentations were applied to the joints. On the next day the pains were less, and the pulse had fallen to one hundred and twenty; but he had not slept. The opium was therefore increased to a grain and a half every third hour.

On the 21st he had slept some, and the pulse had fallen to one hundred and four. The pains were greatly diminished, and the swellings of the joints somewhat lessened. The opium was continued in the same dose. Next day he was found to have slept well, and the pulse had fallen to ninety-two. The opium without the calomel was continued for three days longer in the dose of a grain and a half every third hour, at the end of which period, being quite free from pain, and with a quiet pulse, he was put on bark.

Dr. Corrigan attended his friend Dr. Aldridge, who had a very severe rheumatic attack of three day's duration when he was first seen by Dr. Corrigan. Nearly all the large joints were swollen and acutely painful, and the pains were shifting from joint to joint. The pulse was one hundred and twenty; the tongue very foul, but moist; and the want of rest from the agony of the pain was most distressing. He was immediately put on the opiate treatment. He first got one grain every two hours; the quantity was then increased to one grain every hour; and this was continued for thirteen days, with the administration of an occasional purgative. On the fourteenth day he began to take the *Mist. Guaiaci c. Sulph. Quinæ;* and on the fifteenth day he was walking about his parlour, complaining only of not being so strong as usual, but free from pain and swelling. Dr. Aldridge calculated he had taken about two hundred grains of opium during the treatment.

Mr. H. aged 26, had suffered for three days under a very acute attack of rheumatism. The shoulders, wrists, and knees were swollen, and very painful; and the pains had been so severe that for three nights he had not closed an eye. His pulse was one hundred and twenty, and full; and his tongue moist. He was put on the opium treatment, and took every day for six days from eight to ten grains of opium. On the seventh day, he began to take along with the opium the *Mist. Guaiaci c. Sulph. Quinæ*, and on the eighth day of attendance, he only complained of some stiffness in the affected joints. His pulse had fallen to seventy-six, and his appetite was good.

In one of his cases pericarditis was threatened, the pulse became affected, oppression and constriction across the chest were felt, the countenance became anxious, and the patient was bathed in profuse perspiration. These unpleasant symptoms subsided under the steady use of the opium combined with a grain and a half of quinine to each dose.

Dr. Corrigan remarks, that "the most important rule to be remembered in employing opium for the cure of acute rheumatism is, that full and sufficient doses shall be exhibited;" unless carried to this length it is sure to end in disappointment. He mentions it as a singular circumstance, that sometimes during the progress of the cure under the opium, diarrhœa should come on so as to require the exhibition of chalk and kino. He recommends the local application of flannel soaked in warm spirit of turpentine, or in camphorated spirit, or in simple decoction of poppy heads, to the inflamed surfaces. If the patient should suffer much from constant profuse perspirations, he recommends the conjunction of sulphate of quinine with the opium.

Dr. Corrigan thinks this plan of treatment superior to all others, because it shortens the duration of the attack, alleviates the sufferings of the patient, husbands the strength, and prevents the complications of endocarditis, pericarditis, &c., which so often complicate this disease, and are the cause of prolonged suffering ending only in death.—*Edinburgh Medical and Surgical Journal, from Dublin Journal of Medical Science*, November, 1839.

[*Note.*—Several years ago we had an opportunity of testing the efficacy of this practice, and found it superior to every other. Large doses of opium were at first given, not with the view of curing the disease but of alleviating the intense suffering under which the patient laboured. Since then we have employed it in several cases, and always with the most marked benefit; the disease seldom lasting beyond a fortnight, and being much alleviated during the whole course of the treatment.—ED.]

---

*On a peculiar affection of the Uvula.* By EDWARD THOMPSON.—Mr. Thompson describes the affection to consist of an elongation of the mucous coat of the uvula produced by the effusion of lymph, (serous fluid ?) into the submucous cellular tissue; this elongation sometimes going to the length of two inches. The fluid contained within the expanded mucous coat is of a light amber colour, and so pellucid that the handle of a tea-spoon is readily seen if placed behind it. The body of the uvula itself appears slightly enlarged, and of increased redness, as well as the palatine arches; but the tonsils are not usually affected. He considers the alteration to be the result of active inflammation of the uvula.

The attendant symptoms are those of simple sore throat in the first instance, but afterwards a huskiness of voice, with occasional brief loss of it, are remarked. Soon a distressing feeling of suffocation comes on, and, as the elongation goes on, the patient runs great risk of being suffocated from the pendulous portion passing into the larynx. This in fact is the chief danger, and the patient sometimes falls down with every symptom of suffocation. When the throat is examined the pellucid elongation is very apt to be overlooked, as the extremity of the body of the uvula is seen red and somewhat swollen. The treatment is very simple when the malady is discovered; a simple incision with the lancet, or a cut with a pair of scissors, or even entire removal of the pendulous portion, if large, being all that is required. The membrane usually collapses after the removal of the fluid, and a very small opening is sufficient in most cases for its discharge. —*Ib., from Trans. Prov. Med. and Surg. Association*, Vol. vii.

---

*On the existence of Syphilis in Animals.* By Dr. PAULI of Landau.—Dr. Pauli has recorded two cases which appear to prove that syphilis exists in animals. A veterinary surgeon brought him the penis of a bull on which was a condyloma of the size of a walnut, similar in all respects to that of the human subject. All the cows which this animal had covered were attacked with a mucous running, which generally stopped after a few weeks, but which sometimes required the employment of astringent injections. Another bull infected in a similar manner the cows which it covered; and it was afterwards discovered that it had a similar condyloma of the size of a chestnut on the anterior part of the penis. The cows in this case recovered spontaneously.—*Ibid, from Schmidt's Jahrbucher*, March, 1839.

*On the Employment of Sea Salt (Chloride of Sodium) in Pulmonary Consumption, Scrofulous affections, &c.* By M. AMÉDÉ LATOUR.—M. Latour was first induced to give a trial to this remedy in phthisis, from its reported efficacy in preventing or curing pulmonary complaints among the lower animals. A great mortality prevails amongst the apes and monkeys confined in menageries, chiefly from pulmonary complaints; and the proprietor of a menagerie found, that by the free use of sea salt, he was enabled to preserve these animals in health for seven or eight years; and, even after a cough had manifested itself, the administration of the salt was followed by a rapid cure.

M. Latour relates three cases in the human subject, in which the administration of salt appears to have been followed by the happiest results. In one of the cases, the disease had gone so far, that there was distinct cavernous rattle, with pectoriloquy, muco-purulent and purulent expectoration streaked with blood, great emaciation, hectic fever, &c., and yet the patient made a perfect recovery at the end of a few months, the sea salt having been given uninterruptedly for sixty days.

M. Latour directs a particular regimen to be followed during the treatment. The aliment should consist almost exclusively of beef or mutton grilled or roasted, of good rich soups, or animal jellies. The patient should partake of these in small quantity at a time, but often, and should drink a little good old wine diluted with water. Every fine day, when the sun shines, and during its warmest period, the patient should take gentle exercise in the open air; and his chamber should be well aired twice or thrice a-day. Flannel is recommended to be worn next the skin.

The mode of administration of the salt is as follows: half a drachm to a drachm of the chloride of sodium is administered daily, either in a glass of beef-tea, or in some pectoral infusion, or, if this should excite cough, it may be given in divided doses made up into bread pills, drinking a little beef-tea afterwards. It is best to commence with small doses, as the sudden introduction into the system of such a powerful stimulant, is apt to be followed by congestions of blood in the digestive organs or lungs. A few cresses are recommended to be eaten once or twice every week, after having been well sprinkled with common salt, but no vinegar or oil is allowed with them. To relieve the pains in the chest, and the burning sensations of which the patient complains, instead of the usual pectoral drinks, he prescribes the following: Carrots are to be well boiled in a moderate quantity of water; they are then to be well beaten, and passed through a sieve. The fluid which passes through is then mixed with fresh milk, sweetened with a small quantity of sugar, and flavoured with orange-peel. This compound the patient drinks at his own discretion. In general some thirst is at first caused by the administration of the sea salt, and for this, M. Latour directs a weak infusion of gentian flavoured with orange-peel.—*Ibid, from Gazette des Medecins Practiciens,* 1839.

---

*Successful treatment of Aortic Aneurism by Acetate of Lead.* By MM. DUSOL and LEGROUX.—Aortic aneurism has been always considered to be an incurable affection, the only cases of cure known being the result of natural causes. The treatment generally pursued, viz., oft-repeated bleedings and starvation, evidently increase the serosity or watery parts of the blood, diminish its coagulability, and prevent the formation of those fibrous clots on the formation of which the cure depends. Dupuytren was amongst the first who recommended and employed the acetate of lead in this disease, and his success induced a few other practitioners to give it a fair trial. The results of these trials, MM. Dusol and Legroux have laid before the public. Three cases are recorded at length, but the symptoms and treatment are so similar in all that one will suffice as an example.

Pecheur, 37 years of age was admitted into the wards of M. Dupuytren, in the Hotel-Dieu, on the 12th May, 1829, with a pulsating tumour on the upper and right side of the sternum. Three years before, when lifting a heavy piece of wood, he experienced a sudden attack of pain with difficult respiration in the right side of his chest. He continued to work, however, for fifteen months, but the oppression in the region of the chest augmented, and was attended with violent headach, acute pain in the right shoulder, and right side of the neck, and along the course of the vessels of that region. For this he was bled, but without much relief. After a few months, a tumour appeared on the thorax, which gradually augmented in volume till it acquired the size of an egg, when it became stationary. In proportion as the tumour augmented in volume externally, the dyspnœa diminished; but its recurrence forced the patient to apply for relief at the Hospital. The pulsation of the tumour was perfectly synchronous with that of the pulse; the skin which covered it was red and stretched. There was considerable cough and much dyspnœa, and the patient was obliged to maintain the sitting posture. There was facial congestion, difficult deglutition, and frightful dreams, but the appetite was pretty good and the bowels were regular. Blood-letting to the extent of seven or eight ounces having afforded no relief. M. Dupuytren ordered two pills, each containing one grain of the acetate of lead. On the following day, and every day after, he took six pills; and from this moment amelioration of all the symptoms took place, so that by the 1st June the tumour had almost completely disappeared, and the other symptoms were much relieved.

The number of pills was gradually increased to ten daily, and compresses dipped in a saturnine lotion were applied over the tumour. This treatment was continued till the 29th June, when it was discontinued, from its exciting nausea and vomiting. It was again renewed on the 4th July, and continued till the 19th, when the patient left the hospital, feeling himself quite well.

The amelioration in the three cases related was so remarkable and rapid, that it cannot fail to induce similar trials to be made in this country. Operations on the larger vessels near the heart for the cure of aneurism have very generally been unsuccessful, and any thing which could give a chance of life, particularly without undergoing the danger of an operation, should be eagerly adopted.—*Ibid, from Archives Générales de Médecine*, Sept., 1839.

---

PARISIAN HOSPITAL PRACTICE.

*Contusion of the Sciatic Nerve,—Paralysis of Right Lower Extremity,—Salutary Effects of Strychnine.*—At the Hospital Beaujon, in the wards of M. Marjolin, is a man, aged 50, affected with paralysis of the right lower extremity. The history he gives of his case is as follows:—Ten months ago, while going up stairs, he made a false step, and fell on his right side, the buttock striking against the edge of the stair; the fall was partially broken by his having caught hold of the bannisters, but still he fell with great violence; he immediately felt great pain all over his body, but especially in the part; he got up with difficulty, and walked about fifty paces to enter his room, where he flung himself on the bed, and remained there for about an hour; the pain in his right lower extremity all this time was extreme, and he found it impossible to move his right leg; feeling sick, he attempted to rise to call for assistance, but, during his exertions, he rolled off the bed, and fell upon the floor, where he remained some time, not being able to move, and suffering excruciating pain, extending from the buttock down the thigh and leg, until at length, by dint of crying out, he attracted the attention of two men, who entered his room and raised him to his bed.

A surgeon was sent for, who found the buttock of the right side slightly swollen; pressure on the contused spot caused great pain; the right lower extremity was perfectly insensible, and all power of motion lost; but a severe pain ran from the buttock down the limb, as far as the toes, which were forcibly bent.

The patient was bled copiously at the arm, and poultices were applied over the whole of the limb; this pain and swelling continuing, twenty-five leeches were applied to the buttock, and the poultices, as hot as could be borne, continued; inflammation and suppuration; a regular burn took place from the poultices on the inner part of the foot, attributed, by the patient, to their too great heat, but, probably, arising from the lessened resistance, owing to defective nervous supply. This suppuration lasted for some time, and then healed; the region of the buttock soon became greatly inflamed and swollen, but, after fifteen days, this disappeared.

At the end of a month, finding that the pain had not diminished, and that his limb was as insensible and motionless as at first, he determined on entering the hospital, when he presented the following symptoms:—Continued and severe pain, extending from the middle of the right buttock down the back part of the thigh and leg, as far as the extremity of the toes, following the course of the sciatic nerve; impossibility to perform any motion with his limb; insensibility of the foot and leg, as far as the knee; the heel retracted, and the toes forcibly flexed, the great toe mostly; any attempt at extending them producing great pain; the buttock was swollen, and a contused line, about four inches in length and an inch in breadth, extending across the buttock, above the great trochanter, opposite to the spot where the sciatic nerve makes its exit from the pelvis, and transverse to the direction of the nerve, marked the place where the edge of the stair had struck; the leg and foot felt extremely cold and dead, as if a heavy weight had been placed upon them; their temperature was actually diminished, and the weight of the bed-clothes was so disagreeable as to require their being kept constantly raised; the patient had lost a good deal of flesh generally, but the affected limb was not much more wasted than the other; in every other respect he was perfectly well; he was ordered to take, every second day, sulphureous baths, which, as he derived some benefit from them, were subsequently employed every day; he got up every day, and, with the help of crutches, managed to move about the ward for a short time, but he could not rest upon the affected limb or raise it. The first day he rose from bed, the foot became extremely swollen and red, and a sensation of prickling all over it became so severe as to force him to lie down again, when it disappeared; on every successive attempt, the same circumstances occurred, but in diminishing degree, and, finally, he could remain up for a short time; the ankle became swollen every time, but this disappeared during the night.

Matters remained thus for about a month, no diminution in the symptoms occurring, when, without any obvious cause, the inner part of the leg began to inflame; the inflammation became severe, the leg was greatly swollen, and the inguinal glands were affected: by the successive application of leeches, to the number of one hundred, and of poultices, these symptoms were reduced. Shortly after this, about three months from the accident, the sensibility of the leg returned, but to a greater degree than natural, pinching and pricking were much more painful than on the opposite leg, and even the friction of the bed-clothes was unbearable. This morbid sensibility remained for two months, when it also disappeared, and sensation reverted to its natural state in the leg, but the foot still remained insensible, and the power of motion had not returned.

Three blisters had been successively applied, two on the superior and external surface of the leg, and one much larger on the buttock, between the tuber ischii and the great trochanter. They were kept open, and every day a quarter of a grain of strychnine was sprinkled on their surface. Six weeks ago, eight months and a half from the time of the accident, he began to take internally, strychnine, in doses of a quarter of a grain, made up into pills, twice a day. The first pill produced convulsive motions of the lower extremities, which lasted for several hours during the night, but diminished during the day. These convulsive motions became gradually less, and at present, although he continues the use of the strychnine in the same doses, they rarely occur, and only in the affected limb. From the time he began to take strychnine internally, he found himself getting gradually better, the pain diminished, and he recovered in a slight degree the movements of his limb. At present, ten months from the accident, his state is as follows: general movements of the lower extremity are restored, in the recumbent position he can raise the limb, extend and bend the knee, and also make flexion and extension of the ankle; but the bent or retracted state of the toes still remains, so that he moves on crutches, being unable to place his foot flat upon the ground: sensation is also nearly natural, but the leg feels cold, and is subject to tingling and pricking pains; the thigh is somewhat smaller than the sound one, the leg appears larger, but this is owing to œdema, which is still produced in the erect position; although the strychnine has been continued for so long a time no injurious effects have resulted; on the contrary, the system appears to have become tolerant of its agency.

—

*Crepitation of the Sheaths of Tendons.—Case* 1.—A young man, 21 years old, whilst lifting a heavy weight, which required great muscular exertion, felt a severe pain in the lower half of his right fore-arm. The pain, although great, was not sufficient to force him to cease from working, but, on the following day, he perceived that his fore-arm was swollen, and that the movements of flexion of the thumb were extremely painful, especially when he attempted to grasp any object. He contented himself with remaining quiet, and applying poultices to the part. After twelve days, finding that the swelling remained stationary, although the pain had in great part disappeared, he entered the wards of M. Velpeau, at la Charité.

On examination, a circumscribed swelling was found, situated on the lower part of the right fore-arm, extending from the middle of its posterior surface, and crossing the line of the radius, to terminate at the lower extremity of the bone, defining exactly the course of the long abductor of the thumb: on placing the hand on the sheath of the tendon of this muscle, and of the short extensor, and, on bending the thumb, a sound resembling that produced by rubbing together German tinder or Indian rubber, was perceived at each successive flexion.

Compresses, dipped in camphorated spirits of wine, and compression on the part with a bandage were ordered, and in a few days the patient left the hospital completely cured.

This singular affection, which is called by French surgeons crepitating disease of the sheaths of tendons, although not uncommon, had not excited the attention of surgeons before Boyer, who appears to have been the first to notice it. In his "Traité des Maladies Chirurgicales," we find the following words:—"Persons who employ their hands in labourious occupations, are subject to a singular affection of the cellular tissue surrounding the long abductor and short extensor of the thumb. These muscles project more than usual, and when pressed cause to be heard a peculiar noise, which might be confounded with crepitation, and which cannot be better compared than to that produced by rubbing between the fingers German tinder. This sensation is entirely different from true crepitation of fracture, and can scarcely deceive an experienced surgeon."

Notwithstanding Boyer's remarks on the difficulty of mistake, cases have occurred, in which this affection has been taken for fracture, and treated as such. M. Velpeau, in one of his clinical lectures, said, that his attention had been first attracted to this disease by observing a case, which had been treated for several days as fracture of the radius, and which turned out at last to be simply this affection of the sheaths of tendons. He afterwards had frequent opportunities of studying this affection, and, in the first edition of his "Anatomie des Regions," he described it more fully.

The attention of surgeons having been thus fixed, observations of crepitation of the sheaths of tendons became more numerous, its symptoms better known, and its history more clear, but it has been chiefly illustrated by the observations of continental surgeons.

From the fact, that the labouring class of society is mostly subject to this disease, and that it occurs principally to those persons who, from their occupations, are obliged to exercise continually the muscles of those regions in which it appears, it is evident that the principle and chief cause is continued and repeated exercise of the muscles. Thus we find it generally appears among reapers, washerwomen, carpenters, masons, &c., and commonly after severe labour.

*Case* 2.—In the month of June, 1838, a man of a powerful frame came to the consultation of La Pitié for a swelling of the lower part of the fore-arm.

His employment consisted in the unloading of wine barges, and, although of great muscular force, still he had generally found it extremely fatiguing. The evening before his application at the hospital, he had been employed in removing large iron bars: after working for some hours, he felt at the lower part of his right fore-arm a painful sensation, which was at first slight and did not occupy his attention, but towards evening the pain increased, and on the following morning he perceived a swelling, occupying the spot in which the pain had existed.

On examining the swelling, it was found to occupy the place where the pulse is generally felt, or rather more inwards: it was about an inch and a half in length, an inch in breadth, and nearly the same in elevation. The movements of flexion of the thumb were extremely painful, and on placing the hand on the tumour, a sound similar to the rubbing together of an extremely small chain, was felt on each successive flexion: the position of the swelling and the noise, indicated that the disease was seated in the sheath of the tendon of the long flexor of the thumb.

Leeches and poultices were applied, and the next day the pain had almost disappeared. On the third day the patient felt so well, that he wished to leave the hospital; the crepitating noise had disappeared, the swelling had greatly diminished, and the pain was almost well. He was persuaded to remain a few days longer, and the next day, without any evident cause, the swelling had increased almost to its original size; fluctuation was evident; poultices were simply ordered to be continued. During the fifth, and following days, the swelling gradually diminished, and on the twelfth the patient left the hospital completely cured, without the least swelling, and with perfect recovery of the movements of the fore-arm.

In this case violent and continued exertion was the cause of the disease, and the swelling supervened several hours after the first appearance of pain; but in some cases it would seem the effect of sudden and violent effort.

*Case* 3.—Whilst occupied one day with a friend in pulling a heavy boat against a strong current, to surmount which, great exertion was necessary, my friend suddenly ceased rowing, the oar fell from his grasp, and he complained of great pain in his right fore-arm. On examination, I perceived a considerable swelling, corresponding exactly with the position of the long abductor of the thumb. The movements of flexion of the thumb were painful, and on placing the hand on the swelling, crepitation was felt distinctly. The swelling and pain existed for a few days, and finally disappeared without any treatment.

In the above, and the following case, extracted from the "Thesis" of M. Poulain, published in 1835, the disease was evidently owing to forcible exertion, and supervened suddenly.

*Case* 4.—A farrier was beating a hot piece of iron, with a hammer, weighing from ten to twelve pounds, when, having missed his stroke, the hammer, instead of falling upon the iron, struck with its edge the anvil, and rebounded in an oblique direction with great force; he immediately felt a deadening sensation in his hand and fore-arm, nevertheless, he continued working during the rest of the day, but with difficulty. The next day the posterior surface of the wrist, and the lower part of the fore-arm, were slightly swollen: on forcibly extending the fingers backwards, and the thumb outwards, a crackling noise was heard, which increased to such a degree, that the patient became alarmed, and imagined that he had fracture. He immediately consulted a surgeon, who, on examination, found the bones entire. Leeches and topical applications were employed, but it was only after two months that the disease had entirely disappeared.

External violence is another cause.

*Case* 5.—In 1834, a man, 50 years old, entered the wards of M. Lisfranc, at La Pitié, for a blow he had received on the upper and anterior surface of his arm. On examination, the part was found swollen and contused; separating the arm from the body and carrying it outwards caused great pain, and on attempting to make rotation of the shoulder, a strong and evident crepitation was produced, loud enough to be heard by persons standing round the bed; indeed, the crepitation was so evident, that many believed in the existence of fracture of the surgical neck of the humerus, although all its other symptoms were wanting. M. Lisfranc said that he could not pronounce distinctly whether fracture existed, but that it would be more easily determined when the swelling had diminished. Three days after the accident, it was only after repeated trials that a slight crepitation was heard, and after six days it had entirely disappeared. M. Poulain, the reporter of this case, thinks that the disease was seated in sheaths of the long tendon of the biceps.

Although the fore-arm is the region mostly affected, still it may occupy other parts. M. Velpeau, in his clinical lecture, said that he had seen it behind the ankles, in the palm of the hand, in the palmar surface of the fingers; in fact, it may exist in every region where there are tendons and their sheaths.

When it appears in the lower extremities, it generally results from gymnastic exercises, over-walking or dancing, &c.

The chronic and sub-acute kind seems to result from habitual and continued exertion, as before noticed, in washerwomen, &c. The more acute cases frequently occur in individuals who, unused to forcible exertions, are accidentally called upon to make violent and continued efforts.

*Symptoms.*—The principal and pathognomonic symptom is, as may be seen from the foregoing cases, the peculiar grating noise produced by the motion of the tendons in their sheaths, and probably owing to some change in the synovial secretion. This sensation is mostly accompanied by more or less of swelling and by pain increased by motion; if the fore-arm is affected, the patient experiences great pain in closing the hand, and in grasping anything.

The duration of the disease is generally from fifteen to twenty days, and in the generality of cases it disappears gradually under simple treatment, chiefly quietude, and in the latter stage compression, but in some cases, unfortunately, it would seem, according to M. Velpeau and others, that the disease does not terminate so favourably, severe inflammation of the fibro-serous sheaths of the tendons may result from it, and the inflammation may gain the surrounding tissues, and be productive of serious consequences; in these cases it is evident that our treatment must be more vigorous: leeches, and the other antiphlogistic remedies must be employed, but in the generality of cases, rest, and the application of compresses, dipped in camphorated spirits of wine or Goulard water, with compression, are sufficient.—*Lancet.*

---

### ST. THOMAS' HOSPITAL.

*Severe case of Hæmatemesis.—Salivation produced by Bismuth.*—Ann Gales, a person of slender make, aged 35, admitted, under Dr. Burton, November 12, 1839. Was married five years ago, and till then always enjoyed good health, but was never robust; catamenia always regular. Since marriage has lived in much more confined situations, and has had a good deal of mental trouble. She was much reduced by uterine hæmorrhages at the birth of her first child; the lochia were succeeded by a pale and copious discharge, which continued for three months, when she weaned the child, and they ceased. Has since had four miscarriages, the last in December, 1838, which was attended with much flooding, and she has never quite recovered. She soon became subject to sudden and violent attacks of pain in the epigastrium, which continued till vomiting took place of about half a pint of clear yellowish fluid; this occurred every two or three days; catamenia continued at regular intervals, and of natural colour, but much too abundant in quantity; gradually lost strength and became pale and emaciated, with a good deal of nervous excitability, bursting into tears on the least agitation. Nine weeks before admission, first observed blood in her dejections, it was florid, and voided after some tenesmus; it was apparent every time she went to stool, for nearly a month; when, at the time she was expecting the menses, she took two strong purging pills, which brought away copious, black evacuations. Next day she felt better, but on making some unusual exertion, was seized with the same kind of spasmodic pain which had usually preceded the pyrosis. She fainted, and, on coming too, vomited upwards of three pints of blood, dark and coagulated. In ten days she again fainted under nervous excitement, and vomited half a pint of blood; after this, was repeatedly fainting, and supported by biscuit soaked in wine; violent pulsations of the heart, and great throbbing in the head, followed the slightest motion, and the stools continued quite black.

On admission, there was great general debility, coldness of the extremities, and extreme pallor; pain, on pressure, in the epigastrium, and right hypochondrium; feels very sick and faint when erect, and there is vertigo, tinnitus aurium, and dimness of sight; sleeps pretty well; pulse 120, quick and small; considerable pulsation in the epigastrium, which first appeared with the re-action which followed the first hæmorrhage, and recurred on tho slightest exertion; sounds of heart heard over a larger space than usual; very large at apex, and accompanied by a soft bruit; bowels open; urine sometimes abundant, at others scanty. Ordered milk diet.

*Diluted hydrocyanic acid*, 3 minims;
*Syrup of white poppies*, 1 drachm;
*Camphor mixture*, 1 ounce. To be taken every six hours.

*Nov.* 15.—Feels rather better, but there is considerable tenderness and pain in the epigastric region; a blister was applied, which afforded much relief; she continued to improve, and on the 29th was ordered dry diet with beef tea; the catamenia appeared December 3, in natural quantity.

*Dec.* 6.—Is much better; has continued the draught regularly.

*Trinitrate of bismuth*, 5 grains;
*Dover's powder*, 5 grains. To be taken with the draught three times a day.

7.—Was troubled in the morning with a good deal of heat in the stomach, irregular, fluttering action of the heart, and vertigo. The gums and mouth then became sore, and she began to spit a good deal. The bismuth was stopped, and a drachm of tincture of columba, and half a drachm of aromatic spirit of ammonia ordered with each draught.

10.—Has spit half a pint daily since the 7th; gums sore, slightly tumefied, and edges red; no mercurial fœtor.

Leave off the medicine.

*Sulphate of magnesia*, 1 drachm;
*Diluted sulphuric acid*, 12 minims;
*Infusion of roses*, 1½ ounces. To be taken three times a day.

18.—Much better; pulse 96, fuller; appetite good; sleeps well; no pain.

The last draught to be taken every morning.

Is now convalescent.

—

*Hæmatemesis.*—Louisa Gray, age 23, said to be a house-maid, admitted under the care of Dr. Burton, September 23, 1839. Had been in bad health for the last six months, suffering from hypochondriasis; gradually lost strength and appetite; pulse 120, small and weak; action of heart more extended than usual, with bruit at upper part of sternum; lungs healthy; boils on various parts of body; cicatrices of old ulcers in the groin, &c., yellow coloured discharge from the vagina; catamenia said to be regular. She took digitalis, was bled twice; had blisters applied to the right side and sternum. She was not getting better, and in October was ordered—

*Calomel*, 2 grains;
*Dover's powder*, 2½ grains. To be taken every six hours.

*Oct.* 8.—Bled to fourteen ounces; put on dry diet with beef tea. Considerable tenderness still remaining in the epigastric region; was again bled to twelve ounces on the 11th.

12.—After feeling pain, with a sensation of weight and heat, referred to the epigastrium, for about three hours, she vomited half a pint of fœtid bloody fluid, but which did not coagulate; the same thing took place on the 16th, 19th, and 26th. She was cupped to twelve ounces on the 15th; on the 22d a blister was ordered, and

*Extract of henbane*, 5 grains;
*Ipecacuanha pill*, 1 grain;
*Calomel*, 1 grain. Three times a day.

The sulphate of iron had been given in extract of conium, without good effect, and she was taking four ounces of wine daily.

26.—Ten leeches applied to the groin.

29.—Pulse 126, small; appearance still florid; threw up about a pint of fluid resembling menstrual discharge, at 3 in the morning, and the same quantity at 8 P. M., all at one effort, with some mucus, but no food; bowels opened twice in twenty-four hours, without appearance of blood in the evacuations.

*Dec.* 1.—Threw up half a pint, thicker than before, and clotted; pulse 100, feeble; catamenia came on at night.

3.—Twelve ounces were thrown up, with an odour of port wine. The wine was, therefore, omitted, and a draught was ordered to be taken three times a day, with

*Tincture of sesquichloride of iron*, 15 minims;
*Iodide of potassium*, 3 grains;
*Mint water*, 1½ ounce, being a very convenient and elegant mode of giving the iodide of iron.

4th, 6th, 7th, 9th.—About half a pint on each day; small doses of oil of turpentine were ordered, but did not agree; she was bled again to twelve ounces on the 6th, and ordered sulphate of magnesia, in infusion of roses.

9.—Twenty-five leeches to epigastrium, as pain had become more constant and severe; there was also considerable spinal tenderness in the dorsal region. From 9th to 16th brought up about a teacupful daily.

17.—Ordered milk diet, and a pound of ice to be swallowed daily; croton oil to be applied to the epigastrium, and a turpentine enema every night.

Considerable interest was excited by this case, as, notwithstanding the great loss of blood by the venesection, leeches, and discharge, she gained rather than lost flesh, and though she professed to take nothing but the milk, the evacuations were solid and copious. On being narrowly watched, it was found that one of the nurses supplied her with food, and both were accordingly discharged; that the blood was vomited, there is little doubt, as she was seen frequently ejecting it, and some of the gentlemen found that the clotted appearance was, probably, the effect of the chemical action of the gastric acid, as a dilute hydrochloric acid, mixed with blood, produced the same appearance.—*Lancet.*

---

*Watery solution of Opium in venereal excrescenses.*—M. Venot, of the Veneral Hospital, Bordeaux, having been disappointed in the various remedies which he had employed for the treatment of venereal vegetations, determined to try the efficacy of the narcotic lotions, recommended by M. Desruelles. His experiments were most successful, and from them he draws the following conclusions:—

1. The solution of opium should be fresh and concentrated, an ounce of water containing at least one drachm and a half of opium.

2. The white dry epidermoid vegetations do not yield so readily.

3. All cases of mucous vegetations, moist warts, condylomata, &c., are almost certainly cured by the watery extract of opium, especially if employed after general treatment.

4. The local action of the remedy is manifested in the following manner:—the vegetations dry up, become pale, then yellow, brown, and finally waste away.

5. This action, which is evidently poisonous, may extend to the healthy parts and determine certain accidents, against which the physician must be on his guard.—*Lancet, from Gaz. Med. de Paris*, Jan. 13, 1840.

